# Screening for Depression in Daily Life: Development and External Validation of a Prediction Model Based on Actigraphy and Experience Sampling Method

**DOI:** 10.2196/22634

**Published:** 2020-12-01

**Authors:** Olga Minaeva, Harriëtte Riese, Femke Lamers, Niki Antypa, Marieke Wichers, Sanne H Booij

**Affiliations:** 1 Interdisciplinary Center Psychopathology and Emotion regulation (ICPE), Department of Psychiatry University Medical Center Groningen University of Groningen Groningen Netherlands; 2 Department of Psychiatry, Amsterdam UMC, Amsterdam Public Health Research Institute Vrije Universiteit Amsterdam Netherlands; 3 Department of Clinical Psychology, Institute of Psychology Leiden University Leiden Netherlands; 4 Interdisciplinary Center for Psychopathology and Emotion regulation, Department of Developmental Psychology Faculty of Behavioural and Social Sciences University of Groningen Groningen Netherlands; 5 Center for Integrative Psychiatry, Lentis Groningen Netherlands

**Keywords:** actigraphy, activity tracker, depression, experience sampling method, prediction model, screening

## Abstract

**Background:**

In many countries, depressed individuals often first visit primary care settings for consultation, but a considerable number of clinically depressed patients remain unidentified. Introducing additional screening tools may facilitate the diagnostic process.

**Objective:**

This study aimed to examine whether experience sampling method (ESM)-based measures of depressive affect and behaviors can discriminate depressed from nondepressed individuals. In addition, the added value of actigraphy-based measures was examined.

**Methods:**

We used data from 2 samples to develop and validate prediction models. The development data set included 14 days of ESM and continuous actigraphy of currently depressed (n=43) and nondepressed individuals (n=82). The validation data set included 30 days of ESM and continuous actigraphy of currently depressed (n=27) and nondepressed individuals (n=27). Backward stepwise logistic regression analysis was applied to build the prediction models. Performance of the models was assessed with goodness-of-fit indices, calibration curves, and discriminative ability (area under the receiver operating characteristic curve [AUC]).

**Results:**

In the development data set, the discriminative ability was good for the actigraphy model (AUC=0.790) and excellent for both the ESM (AUC=0.991) and the combined-domains model (AUC=0.993). In the validation data set, the discriminative ability was reasonable for the actigraphy model (AUC=0.648) and excellent for both the ESM (AUC=0.891) and the combined-domains model (AUC=0.892).

**Conclusions:**

ESM is a good diagnostic predictor and is easy to calculate, and it therefore holds promise for implementation in clinical practice. Actigraphy shows no added value to ESM as a diagnostic predictor but might still be useful when ESM use is restricted.

## Introduction

Depressive disorders represent a major public health concern as they are the most prevalent psychiatric disorders and a leading cause of disability worldwide [1,2]. In many countries, depressed individuals often first visit primary care settings when they seek help [3]. Even though most nondepressed individuals can be accurately excluded in primary care [4], a considerable number of clinically depressed patients remains unidentified [5]. Thus, general practitioners can correctly identify between 41.7% and 53.0% of cases of depression with a sensitivity between 41.3% and 59.0% and a specificity between 74.5% and 87.3% [4]. An additional challenge in the detection of depression arises because patients often present with undefined or somatic illness [6], resulting in depression going undetected and often untreated for a longer time period [7].

According to a meta-analysis on the clinical diagnosis of depression in primary care, the accuracy of identification of depression can be improved by prospective examination over an extended period [4]. Therefore, introducing additional screening tools that allow continuous monitoring during daily life may facilitate the diagnostic process and improve referral of depressed individuals to the right care providers. A good candidate for a screening tool that holds particular value for studying mood disorders is the experience sampling method (ESM) [8]. Most commonly delivered via a smartphone, ESM involves repeated, intensive sampling of respondents' current affect, experiences, and behaviors while they are engaged in their daily activities, in their natural environments [9]. Hypothetically, this might be an optimal way of detecting depression risk, as a person can repeatedly assess his/her affect and behaviors in daily life with minimal retrospective recall bias [8]. Previous studies have shown that higher levels of negative affect, which is commonly assessed with ESM, are strongly associated with depression [10]. Furthermore, this method comes closest to the advised method to do longitudinal assessments of depressive symptoms [11,12]. A problem with ESM, however, may be that it places too high a burden on the patient, leading to reduced compliance [9]. Hence, ESM as a screening tool may not be suitable for everyone, warranting the exploration of more passive ways of collecting data as well.

A potential candidate for depression screening that involves passive data collection is ambulatory assessment of actigraphy data from sensors, such as activity trackers. Such activity trackers are now widely used, and they provide ecologically valid data about behavior [13-16]. The data derived from activity trackers include patterns of sleep, physical activity, and circadian rest-activity rhythm (RAR). Alterations in these patterns have been found in depression [17-22] and contributed to objective differentiation of depression subtypes [23]. Further, these behavioral parameters are easily and passively measurable by actigraphy and do not require any invasive procedure or active participation from individuals. Therefore, they do not create an additional burden for an individual [15]. However, it is important to note that the alterations in these actigraphy patterns are not specific for depression, since altered sleep, physical activity, and RAR are present in many other health conditions [24,25]. Therefore, most likely, actigraphy may only be used in addition to other measures when screening for depression. However, the predictive value of actigraphy-based measures, alone and in combination with other measures, for depression remains to be examined.

Sleep, physical activity, and RAR domains are associated with each other [26,27]. However, previous researchers who found associations between actigraphy data and depression included measures from only 1 or 2 of these domains (ie, studying physical activity only, sleep only, or circadian RAR and sleep) [20,21,28-30], with a rare exception [31]. Therefore, probably not all actigraphy-based measures that have been previously associated with depression will have a unique predictive value as part of a multidimensional screening tool. Thus, it is currently still unclear which combination of actigraphy measures are most strongly associated with depression risk.

Using both ESM and actigraphy approaches together for continuous and everyday monitoring of behavioral and affective aspects in depression could be a promising screening tool. While it is assumed that self-reports of depression-related affect and behaviors as assessed with ESM predict depression better than behavioral sensor data, this assumption has never been tested before. To our knowledge, there is only 1 recent study that attempted to predict depression by using both ESM and actigraphy data; however, it was focused on the elderly, had a smaller sample size (N=47), and had no external validation [32]. Currently, it is not yet clear how these approaches perform and if they can be used for screening purposes, both separately and in combination.

In this study, we examined (1) whether ESM-assessed depression-related affect and behavior could discriminate between depressed and nondepressed individuals, (2) whether actigraphy data could discriminate between depressed and nondepressed individuals, and (3) whether actigraphy has added value with respect to the use of ESM. Therefore, we compared the performance of the prediction models with ESM only and actigraphy only to assess the added benefit of the individual domains and then evaluated the performance of the prediction model with both domains included. First, we hypothesized that ESM measures would have a better discriminating ability in distinguishing individuals with and without a diagnosis of depression than actigraphy measures. Second, adding actigraphy measures to the ESM prediction model would improve the discriminating ability. To test these hypotheses, we used 2 data sets for development and validation of the prediction models.

## Methods

### Study Population

We used data from the Netherlands Study of Depression and Anxiety (NESDA) [33] to develop prediction models, and data from the Mood and Movement in Daily Life (MOOVD) study to validate them [34].

#### Development Data Set

In short, NESDA is an ongoing multisite longitudinal cohort study among 2981 adults (aged 18 to 65 years) at baseline, including individuals with depressive and/or anxiety disorders and healthy control subjects, which were recruited from the general population. Details about the total NESDA sample are provided elsewhere [33]. In this study, we used a subsample from the Ecological Momentary Assessment and Actigraphy (EMAA) substudy, which combined 14 days of ESM (5 times a day) with continuous actigraphy [31,35]. A flowchart of the inclusion process for this study is provided in Multimedia Appendix 1. Individuals with a diagnosed episode of major depressive disorder and/or dysthymia in the past month (n=43) and individuals with no lifetime depressive or anxiety disorder (control group, n=82) based on the Composite International Diagnostic Interview (CIDI) [36] were included in the study. Severity of depressive symptoms was assessed with the self-reported Inventory of Depressive Symptomatology (IDS-SR) [37]; the mean IDS-SR score represents moderate depressive symptoms.

#### Validation Data Set

The MOOVD study is an ambulatory assessment study among matched depressed and nondepressed individuals (n=54; aged 20 to 50 years) [34]. Depressed individuals were recruited from 3 psychiatric outpatient centers; nondepressed individuals were recruited from the general population in the Netherlands. This study combined 30 days of ESM (3 times a day) with continuous actigraphy. Depressed individuals (n=27) with a major depressive episode at the time of the interview or within two months prior to the interview, according to the CIDI, were included. Nondepressed individuals (n=27) were free of any mood disorders at the moment of inclusion but were allowed to have a history of depression (n=1, >7 years ago). Severity of depressive symptoms was assessed with the Beck Depression Inventory-II (BDI-II) [38]; the mean BDI-II score represents severe depressive symptoms. Individual scores, however, range from no/mild to severe depressive symptoms in both data sets (IDS-SR score between 9 and 64; BDI-II score between 15 and 51).

### Actigraphy Assessments

NESDA participants wore the wrist-worn GENEActiv accelerometer (Activinsights Ltd) for 24 hours a day for 14 days. GENEActiv validity studies have demonstrated strong correlations for criterion validity (Pearson *r*=0.79-0.98) [39] and a good ability to determine sedentary behavior in adults (aged 18 to 55 years) (Pearson *r*=0.81) [40]. Details of the actigraphy measurements of the NESDA-EMAA substudy are provided elsewhere [31]. MOOVD participants were assessed with an Actical accelerometer (Respironics, Inc) for 24 hours a day for 30 days. In the laboratory study, the Actical demonstrated high reliability (intraclass correlation coefficient=0.92) and validity (*r*=0.81) in adolescents [41]. More information about the actigraphy assessments of the MOOVD study can be found elsewhere [42].

### Experience Sampling Methodology (ESM)

In NESDA, participants took part in the ESM assessment for 2 weeks, during which they filled out questions on smartphones 5 times a day. The electronic diary had a fixed design with 3 hours between each beep, and the questionnaire included items on current mood states, social interactions, daily experiences, and behaviors [35]. Of all ESM assessments of all participants, only 8.3% were missing and all included participants had enough valid data points (>60 time points). In the MOOVD study, participants completed questionnaires on an electronic diary, the PsyMate (PsyMate BV) [43]. The electronic diary had a fixed design with 3 beeps a day, 6 hours apart. The electronic questionnaire contained items about mood, sleep, activities, as well as social interactions, important events, rumination, and self-esteem. Detailed information about the ambulatory assessment procedure is provided elsewhere [34]. All included participants had enough valid diary measurements (>60 time points).

### Outcome Variables

As the main outcome measure for both data sets, we used presence or absence of a diagnosis of depression (major depressive disorder and/or dysthymia) based on DSM-IV criteria [44], assessed with the Composite International Diagnostic Interview (CIDI), version 2.1. The CIDI is a fully structured interview designed for assessing mental disorders according to the diagnostic criteria of the Diagnostic and Statistical Manual of Mental Disorders, fourth edition (DSM-IV) [36]. All CIDIs were performed by well-trained research assistants, mainly psychologists, mental health care nurses, and residents in psychiatry. In the development data set, participants were diagnosed with the CIDI instrument during the regular NESDA interview wave, which was a maximum of 31 days prior to the actigraphy and ESM assessments. In the validation data set, participants started the actigraphy and ESM assessments immediately following the screening CIDI.

### Predictor Variables

Objectively assessed sleep, physical activity, and computed RAR variables, as calculated from the actigraphy data, and ESM-assessed depression-related affect and behavior were used as predictors in our models. Preprocessing of the raw actigraphy data was done in R using GGIR package version 1.5-18 (https://cran.r-project.org/web/packages/GGIR/) for the NESDA data set and described elsewhere [31]. Almost all variables were created similarly in the NESDA and the MOOVD data sets; any exceptions are mentioned.

In the NESDA data set, physical activity was assessed as gross motor activity per day and as minutes of moderate-to-vigorous physical activity (MVPA) per day [45]. Objective gross motor activity was estimated by calculating the Euclidian Norm Minus One (ENMO) per individual per day [31]. Based on those calculations, average estimates of gross motor activity were estimated for each participant. To keep consistent with earlier papers, MVPA was defined as ENMO values greater than 125 mg [31]. In the MOOVD data set, the Actical actigraphy device did not allow the extraction of the raw actigraphy data (data in SI units represented as acceleration in x, y, and z axes), and therefore the data were not processed with the GGIR package. Instead, activity counts (AC) and activity intensity with 4 categories (sedentary, light, moderate, and vigorous) were calculated as a measure of motor activity by an in-built algorithm of the Actical software.

Sleep was assessed as total sleep time (TST) in hours and sleep efficiency per night (%) [21,46]. In the NESDA data set, TST was estimated with the GGIR package and equaled the accumulated nocturnal sustained inactivity bouts. Sleep efficiency was calculated as TST divided by time in bed (estimated by the GGIR package). For the MOOVD data set, TST was calculated as the sum of estimated sleep periods based on the Sadeh algorithm [47]. Sleep efficiency was calculated as a percentage of time scored as sleep during the time spent in bed.

**Figure 1:**
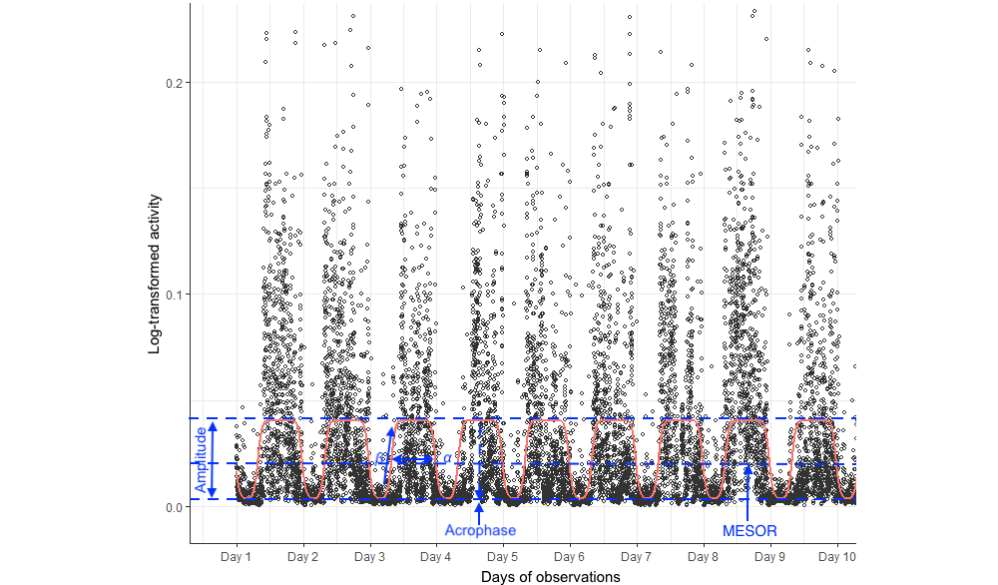
Example of rest-activity rhythm parameters derived from the extended cosinor model. The midline estimating statistic of rhythm (MESOR) is a mean of the modeled activity curve; amplitude is the difference between the peak and trough of the fitted curve, herein estimating the range of activity levels across the 24-hour period; acrophase is a phase marker indicating the time when the fitted curve reaches its peak (ie, time of maximal activity levels across the 24-hour period); α is the relative width of the curve at the middle of the peak; β is an indicator of the steepness of the rise and fall of the curve; and circadian rhythmicity index (F statistic) is an indicator of the strength of circadian rhythmicity (a goodness-of-fit measure for which higher values indicate smaller discrepancies between actigraphy data and values predicted by the cosinor model).

To characterize circadian RAR profiles, individual actigraphy data sets were fitted to an extended cosinor model [48] using nonlinear least-squares regression (RAR package version 1.0.0 for R). This allowed the estimation of 5 circadian curve parameters for each participant, namely the midline estimating statistic of rhythm (MESOR), amplitude, acrophase, α, and β, as well as the circadian rhythmicity index (*F* statistic) (see Figure 1 for more details).

To assess depression-related affect and behavior, we selected ESM items that, in terms of content, matched DSM-V diagnostic criteria for depression. For example, the symptom of “sad or depressed mood” could be represented by the momentary affect state, “I feel sad.” The following items that were present in both data sets were included: sad or depressed mood, irritation, appetite, energy, tiredness, loss of interest, enthusiasm, guilt, concentration, and sleep disturbances. A complete list of included ESM items from the NESDA and the MOOVD data sets can be found in Multimedia Appendix 2. All items were scored on 7-point Likert scales. The sum score of these items calculated for each day and then averaged across 14 days represented depression-related affect and behavior. The person-level Cronbach α for depression-related affect and behavior was .928 in the NESDA data set and .936 in the MOOVD data set. Gender, age, and education level were included in the analysis as covariates.

### Statistical Analysis

Multicollinearity for all predictor variables was checked by calculating Spearman correlations and the variance inflation factors (VIFs). Spearman correlations above 0.80 and VIFs above 10 were considered to be indicative of severe collinearity [49,50]. In this situation, 1 of the collinear variables that was the least related to the outcome variable was removed from further analysis. Fractional polynomials were used to check the presence of nonlinear associations of the continuous predictors to the outcome variable. Cubic association was found for sleep duration (TST) and therefore was included as such in the analysis.

#### Building Single-Domain Models

The next step was to build single-domain models for actigraphy and ESM measurements separately. For the ESM model **(1)**, we included the ESM “depression-related affect and behavior” score and covariates (age, gender, and education), as their association to depression has been consistently shown [51,52] and this information can be easily added to a screening tool. The sum score was chosen instead of including the ESM items in the analysis separately, as it was meant to mimic depressive symptoms.


Group status = a_0_ + a_1_sum score + a_2_age + a_3_gender + a_4_education **(1)**

where “a” represents the regression coefficients from the model: a_1_-a_4_ are predictor coefficients and a_0_ is the intercept.

To build an actigraphy model, we used a multivariate backward stepwise logistic regression approach [53]. A baseline actigraphy model included all actigraphy predictors (amplitude, acrophase, α, β, *F* statistic, ENMO, TST, and sleep efficiency) as well as predefined covariates (age, gender, and education). The MESOR was found to be collinear with ENMO and the least related to the outcome variable; therefore, it was removed from the further analysis. Since different physical activity metrics (ENMO and AC) were available for the development and the validation data sets, we standardized ENMO and AC to alleviate a comparison of the actigraphy models in two data sets. In the following steps, we removed the least significant actigraphy variable (with the highest *P* value) and compared the Akaike information criterion (AIC) value to the AIC from the previous model. A significantly smaller AIC indicates a better model. The procedure was repeated until we defined the optimal combination of actigraphy predictors based on the AIC. The regression equation for the final actigraphy model is included below **(2)**:


Group status = a_0_ + a_1_standardized ENMO + a_2_acrophase + a_3_age + a_4_gender + a_5_education **(2)**

#### Building a Combined-Domains Model

A multivariable backward stepwise logistic regression model was performed to examine what combination of predictors (ie, actigraphy, ESM) resulted in the optimal prediction model for distinguishing between depressed and nondepressed individuals. Since we used a backward approach, the baseline model included all predictors with unique information based on the single-domain models (the actigraphy and the ESM models). In the following models, we used a procedure where we removed variables one by one, based on the highest *P* value, and checked every time whether AIC improved until we defined the final prediction model based on the AIC [54]. The regression equation for the final combined-domains model is included below **(3)**:


Group status = a_0_ + a_1_sum score + a_2_standardized ENMO + a_3_age + a_4_gender + a_5_education **(3)**

#### Evaluation and Validation of the Single-Domain and the Combined-Domains Prediction Models

To evaluate the performance of the combined-domains model and the single-domain models, we utilized goodness-of-fit indices and calibration curves and assessed the discriminative ability of the models (the area under the receiver operating characteristic curve [AUC]) [55]. The goodness-of-fit indices and calibration curves evaluate how close the predicted and observed estimates are. The AUC represents the ability of the models to distinguish between patients with and without the depression diagnosis and ranges from 0.5 (by chance) to 1.0 (perfect discrimination). These quality indicators of all 3 models were compared with a basic model with only the covariates and to each other. As suggested in the TRIPOD (Transparent Reporting of a multivariable prediction model for Individual Prognosis Or Diagnosis) statement [56], we performed bootstrapping techniques for internal validation of the model to simulate the performance of the prediction model in comparable patient data sets.

As a next step, we performed an external validation by using the developed single-domain and combined-domains models from the NESDA data set to assess the predictive performance of the models in the validation sample (ie, the MOOVD data set), calculating the discriminative ability reflected by the AUC. The NESDA data set was chosen to be a development data set because it was larger than the MOOVD data set, to minimize the possibility of overfitting while building the prediction model. The MOOVD data set was suitable for the external validation because inclusion criteria and measurements of depression were similar. Again, we compared the final combined-domains prediction model with the actigraphy and ESM models in the MOOVD data set to check whether it still had a better fit than the single-domain models. The results were reported according to the TRIPOD statement [56].

## Results

The characteristics of the development (NESDA) and validation (MOOVD) data sets are given in Table 1.

**Table 1:** Characteristics of development and validation data sets.

			NESDA^a^ (n=125)				MOOVD^b^ (n=54)				
			Depressed (n=43)		Control (n=82)		Depressed (n=27)		Control (n=27)		
**Study characteristics**											
	Data collection period		2014-2017				2012-2014				
	Setting		General population, primary health care, and mental health care				General population, and outpatient centers for psychiatry				
	Inclusion criteria for cases		Receiving a depression diagnosis 1 month before the ESM^c^/actigraphy assessment				Receiving a depression diagnosis 1 month before the ESM/actigraphy assessment				
	Outcome		Presence or absence of a depression diagnosis (MDD^d^ and/or dysthymia) based on DSM-IV^e^ criteria				Presence or absence of a depression diagnosis (MDD and/or dysthymia) based on DSM-IV criteria				
	Prevalence of outcome, n (%)		43 (34.4)				27 (50.0)				
**Sociodemographic characteristics**											
	Female, n (%)		29 (67.4)		46 (56.1)		20 (74.1)				20 (74.1)
	Age, mean (SD)		52.14 (9.57)		51.50 (12.70)		34.70 (9.86)				34.04 (8.96)
	Education (high), n (%)		13 (30.2)		49 (59.8)		17 (63.0)				19 (70.4)
**Psychopathology**											
	Depression severity instrument		IDS-SR^f^				BDI-II^g^				
	Depression severity, mean (range)		34.53 (9-64)		5.44 (0-25)		31.33 (15-51)				2.26 (0-10)
	AD^h^ and/or BD^i^ use, n (%)		23 (53.5)		4 (4.9)		15 (55.6)				1 (3.7)

^a^NESDA: the Netherlands Study of Depression and Anxiety.

^b^MOOVD: Mood and Movement in Daily Life.

^c^ESM: experience sampling method.

^d^MDD: major depressive disorder.

^e^DSM-IV: Diagnostic and Statistical Manual of Mental Disorders, fourth edition.

^f^IDS-SR: Inventory of Depressive Symptomatology (self-report).

^g^BDI-II: Beck Depression Inventory-II.

^h^AD: antidepressant.

^i^BD: benzodiazepine.

Predictors of depression in the final models were the “depression-related affect and behavior” sum score for the ESM model and gross motor activity (ENMO) and the time of maximal activity levels across the 24-hour period (acrophase) for the actigraphy model (Table 2). The combined-domains prediction model included the “depression-related affect and behavior” sum score and ENMO variables.

For the ESM model, the predictive capacity was 95.2%, which is 29.6% higher than in the null (only intercept) model (65.6%). Calibration of the ESM model was adequate with a Nagelkerke *R^2^* statistic of 0.904 and a Hosmer-Lemeshow goodness-of-fit test of 0.960 (*P*=.998).

For the actigraphy model, the predictive capacity of the final step of the backward selection model was 71.8% (6.2% higher than the null model). Calibration of the actigraphy model was adequate with a Nagelkerke *R^2^* statistic of 0.357 and a Hosmer-Lemeshow goodness-of-fit test of 10.678 (*P*=.221).

The final (combined-domains) model had the same predictive capacity as the ESM model (95.2%). Calibration of the final model was adequate with a Nagelkerke *R^2^* statistic of 0.913 and a Hosmer-Lemeshow goodness-of-fit test of 1.786 (*P*=.987). In all 3 calibration plots, the slope approached the diagonal (see Multimedia Appendix 3).

**Table 2:** Predictors of depression included in the experience sampling method (ESM) prediction model, the actigraphy prediction model, and the final combined-domains model.

Predictor		B^a^		SE		*P* value		Exp(B)^b^		95% CI for Exp(B)
**ESM prediction model**										
	Intercept		–27.880		8.668		.001^c^		0.000	
	Gender		–2.955		1.597		.06		0.052	0.002-1.191
	Age		0.143		0.068		.03^c^		1.154	1.009-1.319
	Education		–1.701		1.058		.11		0.182	0.023-1.452
	Sum score^d^		0.891		0.243		<.001^c^		2.437	1.513-3.923
**Actigraphy prediction model**										
	Intercept		0.050		2.954		.99		1.052	
	Gender		–1.021		0.513		.046^c^		0.360	0.132-0.984
	Age		–0.012		0.020		.54		0.988	0.950-1.027
	Education		–1.711		0.456		.001^c^		0.181	0.074-0.441
	Z-score ENMO^e^		–1.004		0.283		<.001^c^		0.367	0.211-0.638
	Acrophase		0.289		0.177		.10		1.335	0.943-1.888
**Final combined-domains prediction model**										
	Intercept		–25.841		9.314		.01^c^		0.000	
	Gender		–3.751		1.740		.03^c^		0.024	0.001-0.712
	Age		0.102		0.076		.18		1.107	0.953-1.286
	Education		–1.988		1.163		.09		0.137	0.014-1.339
	Sum score		0.919		0.263		<.001^c^		2.508	1.498-4.197
	Z-score ENMO		–1.117		0.767		.15		0.327	0.073-1.472

^a^B: regression coefficient.

^b^Exp(B): exponentiation of the B coefficient (odds ratio).

^c^Significant *P* value (*P*<.05).

^d^Sum score: a person-level sum score of the ESM items that represent depression-related affect and behavior.

^e^ENMO: Euclidian Norm Minus One.

**Figure 2:**
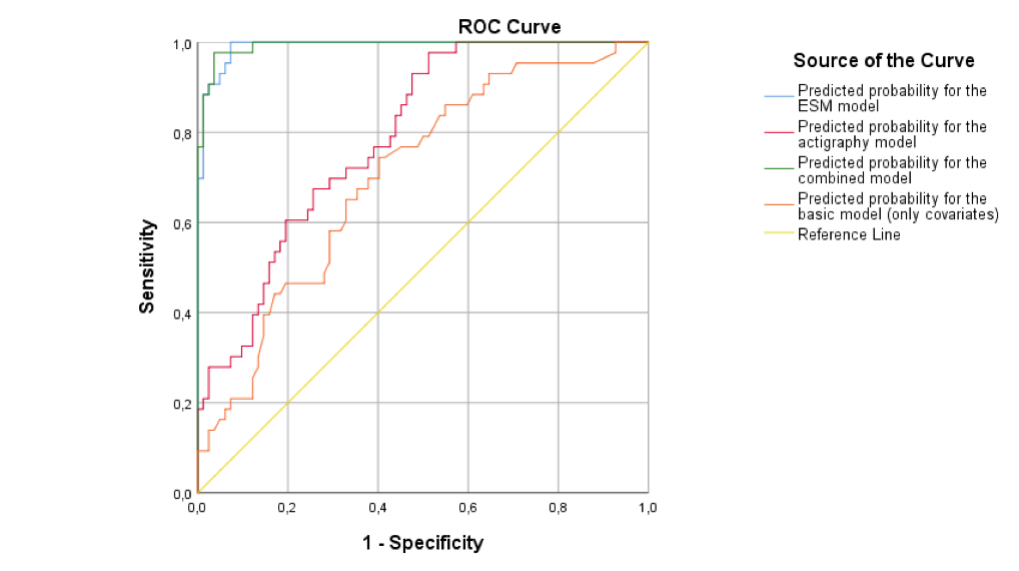
The receiver operating characteristic (ROC) curves of the basic model, the experience sampling method (ESM) model, the actigraphy model, and the combined-domains model in the development data set (the Netherlands Study of Depression and Anxiety).

In the development data set, the discriminative ability in predicting depression was good in the actigraphy model (AUC=0.790) and excellent in the ESM model (AUC=0.991) and the combined-domains model (AUC=0.993) (Table 3, Figure 2). The estimations did not meaningfully change after internal validation. The discriminative ability of the model in the external validation sample was reasonable in the actigraphy model (AUC=0.648) and very good in the ESM model (AUC=0.891) and the combined-domains model (AUC=0.892) (Multimedia Appendix 4). Calibration of all models was adequate, with the slope approaching the diagonal in all 3 calibration plots (Multimedia Appendix 5). Multimedia Appendices 6 and 7 provide an overview of diﬀerent probability thresholds and their respective classiﬁcation measures (sensitivity, speciﬁcity, and predictive values) for all 3 models of interest in the development and validation data sets.

**Table 3:** The discriminative ability of the final combined-domains model and the basic, actigraphy, and experience sampling method (ESM) models.

Models	Development data set (NESDA^a^; n=125)		Validation data set (MOOVD^b^; n=54)		
	AUC^c^	95% CI	AUC		95% CI
Basic model^d^	0.704	0.610-0.798	0.464		0.304-0.624
ESM model	0.991	0.981-1.000	0.891		0.802-0.979
Actigraphy model	0.790	0.713-0.867	0.648		0.492-0.803
Combined-domains model	0.993	0.983-1.000	0.892		0.800-0.984

^a^NESDA: the Netherlands Study of Depression and Anxiety.

^b^MOOVD: Mood and Movement in Daily Life.

^c^AUC: area under the receiver operating characteristic curve.

^d^Basic model has covariates included only (gender, age, and education).

## Discussion

### Principal Results

In this paper, we have developed 3 prediction models based on ESM measures of depression-related affect and behaviors, on actigraphy, and on their combination, for discriminating currently depressed from nondepressed individuals. To our knowledge, this is the first study that has created and compared such models for their individual performance and for their performance in combination, and using both internal and external validation to test their discriminative abilities. The ESM model had an excellent predictive potential in discriminating depressed and nondepressed individuals in both the development and the validation data sets. The actigraphy model, in turn, had a reasonable predictive potential alone but could not compete with the ESM model in predictive performance. The combined-domains prediction model, which included the ESM measure as well as the best combination of actigraphy measures, was very similar to the ESM model in performance in both the development and the validation data sets. Hence, from the results we can conclude that the ESM and actigraphy measures both have the potential to serve as an additional screening tool; however, actigraphy does not have added value when combined with ESM.

### Comparison with Prior Work

The ESM sum score combined items about positive affect, negative affect, sleep, and appetite. Measuring these symptom-related items over a prolonged period of time resulted in successful discrimination between depressed and nondepressed individuals. The constructed prediction model performed excellently, not only in the development but also in the validation data set. In line with our findings, multiple previous studies showed correlations between related measures, mainly negative affect and positive affect (assessed with ESM), and depression [10,12,57]. Another study attempted to estimate depressive symptoms based on the ESM items and found significant correlations with depressive symptoms assessed with symptom questionnaires [11]. Despite the strong correlation between ESM-assessed depression-related affect and behavior and depression, there have been no diagnostic prediction studies using such ESM data. Existing literature, however, indicates that both negative affect and positive affect play a role in predicting relapse of depression [58,59] and future treatment outcome [60,61]. Additionally, negative affect was found to be predictive of depression onset in youth [62]. There are a very limited number of prognostic studies available, focusing on older adults [63]. Authors have identified the items “sad” and “tired” as sensitive measures that have the potential to predict future depression status in older adults. Although replication in other types of samples is warranted, our results suggest that the ESM measure has potential for screening purposes in clinical practice.

Interestingly, self-report measures of depressive affect and behavior largely overperformed objective measures of behavior in distinguishing depressed from nondepressed individuals. Researchers often make the implicit assumption that subjective measures are inaccurate and cannot compete with objective assessments. Self-reported affect and behavior, indeed, might not align with actual observed affect or behavior due to a difference between perceived and actual behavior [64]. Nevertheless, this bias in how a depressed person perceives their own emotions and feelings might be highly useful for predicting depression.

Although the actigraphy model showed good performance in the development data set, its performance dropped in the validation data set. While this discrepancy may be a signal of worse performance in other samples, it could also partly be due to differences in physical activity metrics in use. We found that metrics from the GENEActiv and Actical accelerometers (ENMO and AC, respectively) have never been directly compared before, hence we could not apply any known formula to transform data from one into the other. To overcome this issue, we z-transformed ENMO and AC before the analysis to adjust the variables to the same scale from –1 to 1. This step improved the performance of the actigraphy model, although it was still lower in the validation data set. Of note, it has previously been shown that the placement of the accelerometer device on the body influences its outcome measures in both the level of and fluctuations in activities [65]. Wrist placement was recommended as a basic technique to capture motor activity in depressed patients because it records whole-body movement and gestures [66]. In this study, the devices were worn on the wrist, which implies that the model is only applicable to actigraphy as measured using the wrist placement. Despite the existing challenges and a lower predictive capacity compared with ESM, actigraphy, being a passive data collection method, might still be useful for screening purposes in some cases, for example, in a situation where the use of ESM is not possible or too bothersome for particular individuals.

In agreement with previous studies that used actigraphy data [20-22,31,67], we have found strong associations between lower levels of physical activity and depressive symptoms/depressive disorders. Other RAR characteristics such as MESOR, amplitude, α, β, and *F* statistic, calculated with the extended cosinor analysis, were not sufficiently predictive when assessed together with the overall activity level (ENMO) and therefore, were eliminated from the model. Interestingly, the individual associations between these parameters and depression were significant; however, when these variables were combined, only a few remained in the model (ENMO and acrophase). The fact that only a few variables remained might be due to the potential overlap of these parameters. Even though the association between circadian rest-activity parameters and depression has been shown previously [30,68-71], to our knowledge, there are no studies that specifically compared the predictive ability of various actigraphy-based parameters from different domains in distinguishing depressed and non-depressed individuals. Hence, future studies should further examine to what extent these different variables are measuring partly the same concepts.

The combined-domains model had an excellent performance that was highly similar to the performance of the ESM model. Adding the actigraphy model component (ENMO or AC) did not significantly improve the performance of the combined-domains model. The explanation for this lack of improvement might be that ESM captures part of actigraphy variance. This overlap may be in part because both ESM and actigraphy assess sleep; one assesses subjective sleep duration and subjective sleep quality, while the other assesses the same characteristics objectively. Another example of shared variance might be that complaints like anhedonia could result in a patient being less active and hence, the reduced physical activity may be a result of the depressive symptoms. As in the previous example, concentration problems could be associated with lower sleep quality [72]. This makes questionable the hypothesis of whether actigraphy has added value in its own right when combined with ESM. To our knowledge, there is only 1 recent study that attempted to predict depression by using both ESM and actigraphy data; however, it was focused on community-dwelling older adults, had a smaller sample size (N=47), and had no external validation [32]. These researchers used a wide range of various predictors based on actigraphy and ESM in a machine learning approach to define the optimal combination of the predictors and build a prediction model. As in our study, these researchers developed their model on the basis of daily mean ESM scores, and actigraphy-based daily mean activity levels and daily sleep efficiency variables, although the latter was removed from our prediction models. Hence, the chosen variables showed predictive potential in detecting depression even when different selection approaches were applied. More details of the studies discussed in this paragraph are provided in Multimedia Appendix 8.

### Strengths and Limitations

The main strength of this study was the external validation of the prediction models in a different and adequate data set that prevented overstating the results [56,73]. The fact that there were substantial differences between the 2 data sets and the results were consistent in both data sets provides strong evidence that the prediction models can be generalized to new patients [73]. Finally, all constructed models include variables that are ecologically valid and easy to measure in real life. There are also some limitations of the study that need to be mentioned. First, different physical activity metrics (ENMO versus AC) limited the ability to externally validate the actigraphy prediction model in an optimal way. To avoid this problem in the future, researchers should preferably use the accelerometers that allow access to the raw data. In this case, the same output metric can be chosen so that the algorithms for the computation will be the same or at least comparable. Second, the development data set had a small number of individuals in the depression groups who reported no or mild depressive symptoms. This was most likely due to logistic reasons typical of large cohort studies, as our study was. Participants were diagnosed with the CIDI instrument during the regular NESDA interview wave, which was a maximum of 31 days prior to the actigraphy assessments, whereas depression severity assessment with the IDS-SR questionnaire was not necessarily done close to the actigraphy assessment period (up to 72 days prior and two cases of 74 and 351 days after the CIDI). This situation, however, could possibly deflate the association rather than inflate it, and the model would have more predictive capacity in more acutely depressed individuals. Third, the size of the validation data set was smaller than suggested by some simulation studies, which required at least 100 events for the validation sample. This suggestion, however, was based on limited simulation studies and it lacks the empirical evidence to guide research [56]. The sample size, therefore, is often determined by the available data, as was the case in our study [56]. Finally, both development and validation samples were collected in the Netherlands, which limits generalizability to countries with similar ethnicity profiles and health care systems.

Concerning future research, the constructed ESM models performed excellently with 14 and 30 days of continuous measurements in the development and validation data sets, respectively. However, if a shorter duration of ESM measurements showed similar performance in the prediction of depression, a shorter measurement period could potentially reduce the burden on patients who struggle with longer ESM regimes. Indeed, it has previously been shown that an association between negative affect and depression can already be detected using ESM for 6 to 7 days [60,74,75] or even 3 days [76]. Further, different accelerometer devices can differ substantially in sampling frequency, data processing algorithms, and other characteristics [77]. Therefore, examining various accelerometers and possibly creating formulas for output transformation might be valuable to facilitate the comparison of outputs from different devices. In this study, we assessed 2 samples with currently depressed and nondepressed individuals with moderate to severe levels of depressive symptoms. Therefore, it remains to be seen whether the results of the constructed models generalize to a population with more ambiguous depressive symptoms or other psychiatric problems. A large-scale study in the general population is needed before recommendations can be made for the use of such prediction models in clinical practice. If this is the case, implementation of such a prediction tool in practice can be relatively straightforward. First, ESM seems to be a feasible tool for clinical practice and has the additional benefit that clients become co-owners of the care process [78,79]. Second, the developed algorithm for calculating “depression-related affect and behavior” sum scores is easy to use. However, further automation processes are needed to facilitate use of such a tool in real-life settings, such as primary care. Finally, such studies, including the current one, are necessary to build up empirical evidence on mechanisms, which will be the basis for developing medical devices in the future. Using strict regulations for such kinds of basic research might strangle innovative studies rather than pushing them forward.

### Conclusions

To conclude, in this study we presented models to predict depression based on ESM-assessed depression-related affect and behavior as well as actigraphy data. The most potent predictor was the “depression-related affect and behavior” sum score constructed from the ESM data. The ESM model had an excellent predictive capacity, is easy to calculate, and hence might be feasible for future implementation in clinical practice. The actigraphy model had reasonable performance, but there was no added value of actigraphy in combination with ESM. Despite the fact that the actigraphy model had a lower predictive capacity than ESM, actigraphy could still be of value in situations where ESM is too burdensome.

### Authors’ Contributors

OM, SHB, MW, and HR were involved in the design of the study. OM and SHB performed the statistical analysis. OM wrote the original draft of the manuscript. OM, SHB, MW, HR, FL, and NA critically revised the draft for intellectual content and approved the final version.

## Appendix

Multimedia Appendix 1 Flowchart of a study sample from the Netherland Study of Depression and Anxiety-Ecological Momentary Assessment and Actigraphy (NESDA-EMAA) study.

Multimedia Appendix 2 List of included experience sampling method (ESM) items from the Netherland Study of Depression and Anxiety (NESDA) and the Mood and Movement in Daily Life (MOOVD) data sets and corresponding Diagnostic and Statistical Manual of Mental Disorders, Fifth Edition (DMS-5) criteria.

Multimedia Appendix 3 Calibration plots of the models in the development data set.

Multimedia Appendix 4 The receiver operating characteristic (ROC) curves in the validation dataset.

Multimedia Appendix 5 Calibration plots of the models in the validation data set.

Multimedia Appendix 6 Sensitivity, specificity, and cutoff score for the experience sampling method (ESM) model, the actigraphy model, and the final (combined-domains) model in the development data set.

Multimedia Appendix 7 Sensitivity, specificity, and cutoff score for the experience sampling method (ESM) model, the actigraphy model, and the final (combined-domains) model in the validation data set.

Multimedia Appendix 8 Quantitative comparisons of the articles referred to in the discussion.

## Abbreviations

AC: activity counts
AIC: Akaike information criterion
AUC: the area under the receiver operating characteristic curve
BDI-II: Beck Depression Inventory-II
CIDI: Composite International Diagnostic Interview
DSM: Diagnostic and Statistical Manual of Mental Disorders
ENMO: Euclidian Norm Minus One ESM: experience sampling method
IDS-SR: Inventory of Depressive Symptomatology, self-report
MESOR: midline estimating statistic of rhythm
MOOVD: Mood and Movement in Daily Life
MVPA: moderate-to-vigorous physical activity
NESDA: the Netherlands Study of Depression and Anxiety
NESDA-EMAA: Ecological Momentary Assessment and Actigraphy substudy of NESDA
RAR: rest-activity rhythm
TRIPOD: Transparent Reporting of a multivariable prediction model for Individual Prognosis Or Diagnosis
TST: total sleep time
VIF: variance inflation factor
