# Supplementary material for: Screening for Depression in Daily Life: Development and External Validation of a Prediction Model Based on Actigraphy and Experience Sampling Method
Source: J Med Internet Res. 2020 Dec 1;22(12):e22634. doi: 10.2196/22634 (PMC7894744; doi:10.2196/22634)
Supplement: Multimedia Appendix 1 [file jmir_v22i12e22634_app1.docx]

# **Figure S1. Flow-chart of a study sample from the NESDA-EMAA study**

Participants in the NESDA-EMAA study

(N=384)

No actigraphy data available (N=14)

Reasons:

- technical failure (N=6)

- unknown reason (N=4)

- drop-out (N=2)

- watch lost in mail (N=1)

- watch not correctly set-up (N=1)

Actigraphy data available (N=370)

No suitable data (N=11)

Reasons:

- recording less than 10 days with at least 16 hours (N=9)

- calibration error (N=1)

- data processing did not work (N=1)

Included in the analysis (N=125)

Did not meet inclusion criteria (N=234)

Reasons:

- remitted depression (N=152)

- anxiety only (N=67)

- depression in past 6 months but not in past 1 month (N=15)

Participants in the NESDA wave 6

(N=2069)

Participants in the NESDA wave 6 face-to-face interview (N=1776)

(N=2436)

Newly enrolled NESDA siblings participated in the NESDA wave 6 face-to-face interview

(N=367)

Did not have face-to-face interview

(N=293)

Approached for the NESDA-EMAA study

(N=1017)

Not included (N=633)

Reasons:

- could not be contacted/ no response (N=48)

- could not be included within 30 days (N=132)

- traveling abroad during test period (N=41)

- not interested (N=316)

- no time (N=172)

- too intense (N=117)

- unspecified (N=27)

- will not/cannot use smartphone/actigraphy device (N=37)

- other reasons (N=59)

Not approached (N=248)

Reasons:

- did not give permission (N=128)

- not eligible, presence of a lifetime depressive and/or anxiety disorder (N=120)

Not approached (N=878)

Reasons:

- did not give permission (N=541)

- not eligible, did not participate in at least 2 waves (N=1)

- EMAA study had not started at the moment of the interview (N=336)
