# Supplementary material for: Screening for Depression in Daily Life: Development and External Validation of a Prediction Model Based on Actigraphy and Experience Sampling Method
Source: J Med Internet Res. 2020 Dec 1;22(12):e22634. doi: 10.2196/22634 (PMC7894744; doi:10.2196/22634)
Supplement: Multimedia Appendix 2 [file jmir_v22i12e22634_app2.docx]

# **Table S1. List of included ESM items (in Dutch (English)) from the NESDA and the MOOVD datasets and corresponding DMS-5 criteria**

| **DMS-5 criteria** | **Symptom** | **NESDA ESM corresponding items** | **Range** | **MOOVD ESM corresponding items** | **Range** |
| --- | --- | --- | --- | --- | --- |
| Depressed mood: For children and adolescents, this can also be an irritable mood | Sad mood | Q9. Ik voel me somber (I feel down) | 1-7 (not at all - a lot) | Q19. Ik voel me somber (I feel down) | 1-7 (not at all - a lot) |
|  | Irritation | Q7. Ik voel me geïrriteerd (I feel irritated) | 1-7 (not at all - a lot) | Q16. Ik voel me geïrriteerd (I feel irritated) | 1-7 (not at all - a lot) |
| Diminished interest or loss of pleasure in almost all activities (anhedonia) | Interest | Q8. Ik voel me lusteloos (I feel listless/apathic) | 1-7 (not at all - a lot) | Q7. Mijn interesse in de dingen om me heen is … (My interest in the things around me is …) | -3 – 3 (less than usual - more than usual) |
|  |  | Q11. Ik voel me enthousiast (I feel enthusiastic) |  |  |  |
|  |  |  |  | Q12. Ik voel me enthousiast (I feel enthousastic) | 1-7 (not at all - a lot) |
| Significant weight change or appetite disturbance | Appetite | Q29. Heeft u vandaag meer of minder gegeten dan u doorgaans zou doen? (Have you eaten more or less than usual today?) | 1-7 (much less - much more) | Q5. Mijn eetlust is (My appetite is…) | -3 - 3 (less than usual - more than usual) |
| Sleep disturbance (insomnia or hypersomnia) | Sleep disturbance | Q23a. Heeft u goed geslapen? (Did you sleep well?) | 1-7 (not good - very good) | Q2. Heeft u goed geslapen? (Did you sleep well?) | 1-7 (not good - very good) |
| Psychomotor agitation or retardation | Psychomotor behavior | Not applicable |  | Not applicable |  |
| Fatigue or loss of energy | Energy | Q10. Ik voel me energiek (I feel energetic) | 1-7 (not at all - a lot) | Q9. Ik voel me energiek (I feel energetic) | 1-7 (not at all - a lot) |
|  | Tiredness | Q19. Ik voel me moe (I feel tired) | 1-7 (not at all - a lot) | Q23. Ik voel me moe (I feel tired) | 1-7 (not at all - a lot) |
| Feelings of worthlessness | Guilt | Q16. Ik voel me schuldig (I feel guilty) | 1-7 (not at all - a lot) | Q21. Ik voel me schuldig (I feel guilty) | 1-7 (not at all - a lot) |
| Diminished ability to think or concentrate; indecisiveness | Concentration | Q17. Ik kan me goed concentreren (I can concentrate well) | 1-7 (not at all - a lot) | Q31. Ik kan me goed concentreren (I can concentrate well) | 1-7 (not at all - a lot) |
| Recurrent thoughts of death, recurrent suicidal ideation, or a suicide attempt or specific plan for committing suicide | Thoughts of death | Not applicable |  | Not applicable |  |

Abbreviations. DSM-5 - the diagnostic and statistical manual of mental disorders, fifth edition; ESM - experience sampling method; MOOVD - the mood and movement in daily life study; NESDA - the Netherlands study of depression and anxiety
