# Supplementary material for: Screening for Depression in Daily Life: Development and External Validation of a Prediction Model Based on Actigraphy and Experience Sampling Method
Source: J Med Internet Res. 2020 Dec 1;22(12):e22634. doi: 10.2196/22634 (PMC7894744; doi:10.2196/22634)
Supplement: Multimedia Appendix 3 [file jmir_v22i12e22634_app3.docx]

# **Figure S2. Calibration plots of the models in the development dataset**

**Predicted probability**

**Actual probability**

Best fitting line

R^2^=.953


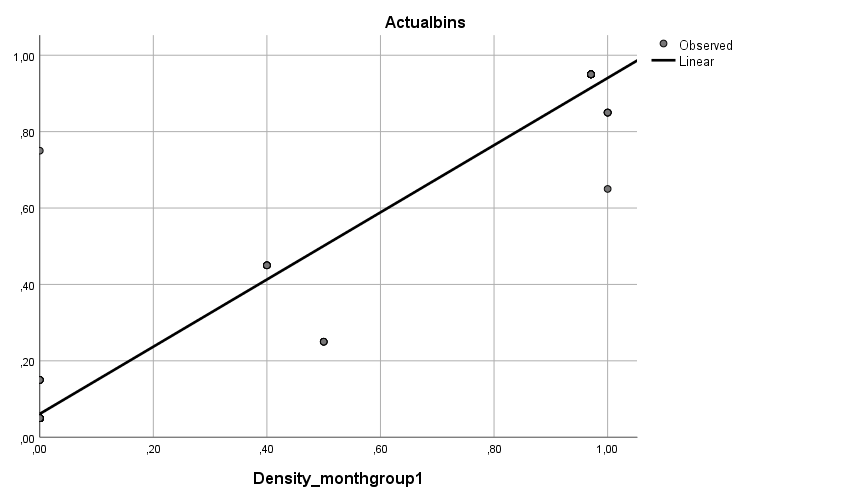


**Figure S2a. A calibration plot of the ESM model in the development dataset**


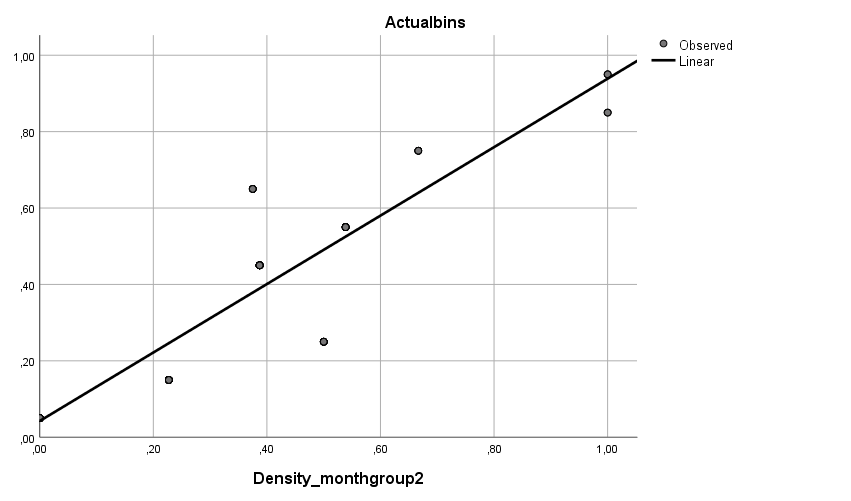


**Predicted probability**

**Actual probability**

Best fitting line

R^2^=.780

**Figure S2b. A calibration plot of the actigraphy model in the development dataset**


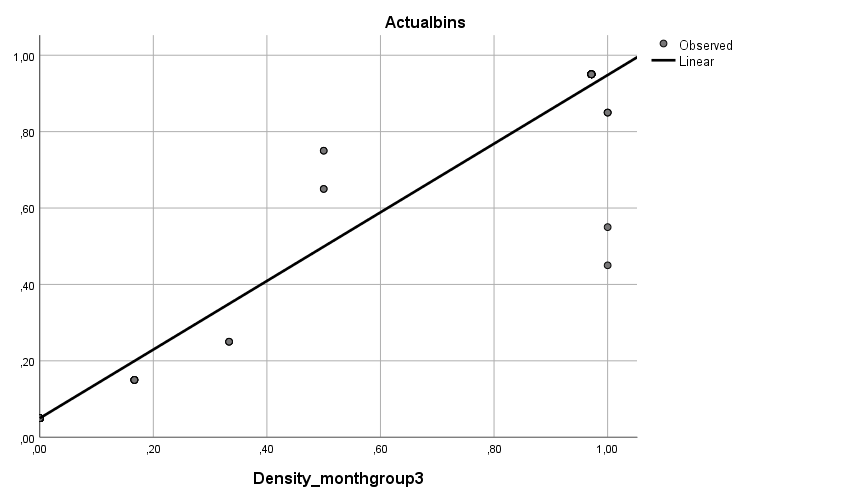


**Predicted probability**

**te**

**Actual probability**

**probability**

**Actual probability**

Best fitting line

R^2^=.967

**Figure S2c. A calibration plot of the final (combined-domains) model in the development dataset**
