# Supplementary material for: Screening for Depression in Daily Life: Development and External Validation of a Prediction Model Based on Actigraphy and Experience Sampling Method
Source: J Med Internet Res. 2020 Dec 1;22(12):e22634. doi: 10.2196/22634 (PMC7894744; doi:10.2196/22634)
Supplement: Multimedia Appendix 4 [file jmir_v22i12e22634_app4.docx]

# **Figure S4. The receiver operating characteristic (ROC) curves**

#
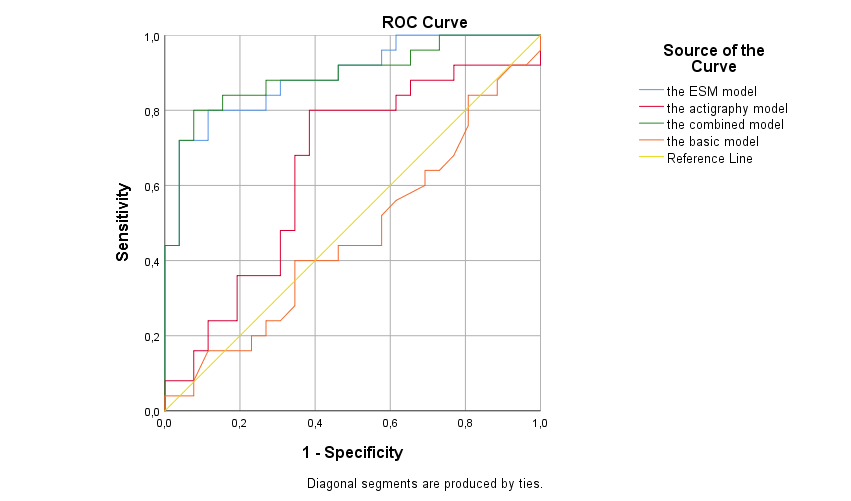
**Figure S4. The receiver operating characteristic (ROC) curves of the basic model, the ESM model, the actigraphy model, and the combined-domains model in the validation dataset (MOOVD).**
