# Supplementary material for: Screening for Depression in Daily Life: Development and External Validation of a Prediction Model Based on Actigraphy and Experience Sampling Method
Source: J Med Internet Res. 2020 Dec 1;22(12):e22634. doi: 10.2196/22634 (PMC7894744; doi:10.2196/22634)
Supplement: Multimedia Appendix 5 [file jmir_v22i12e22634_app5.docx]

# **Figure S3. Calibration plots of the models in the validation dataset**

Best fitting line

R^2^=.866

**Actual probability**

**Predicted probability**


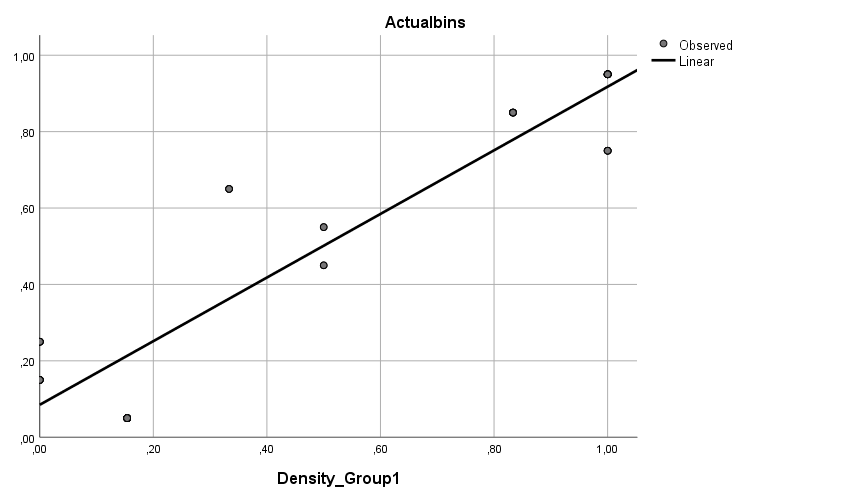


**Figure S3a. A calibration plot of the ESM model in the validation dataset**

Best fitting line

R^2^=.872

**Actual probability**

**Predicted probability**


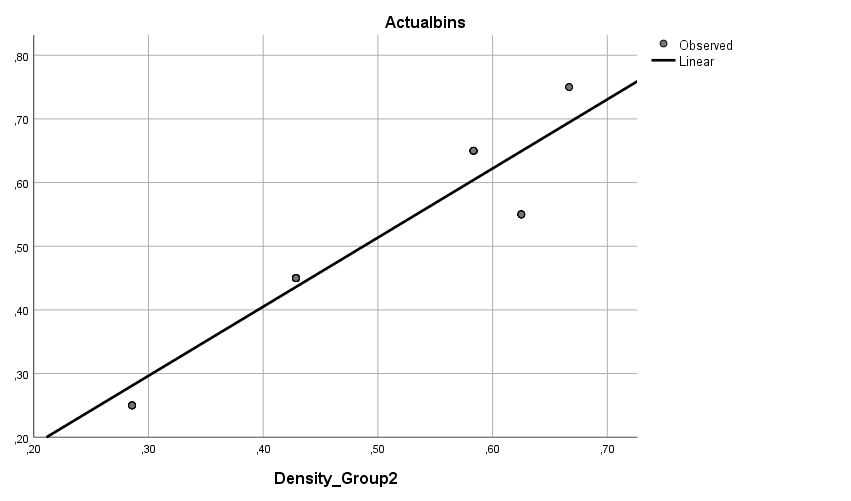


**Figure S3b. A calibration plot of the actigraphy model in the validation dataset**


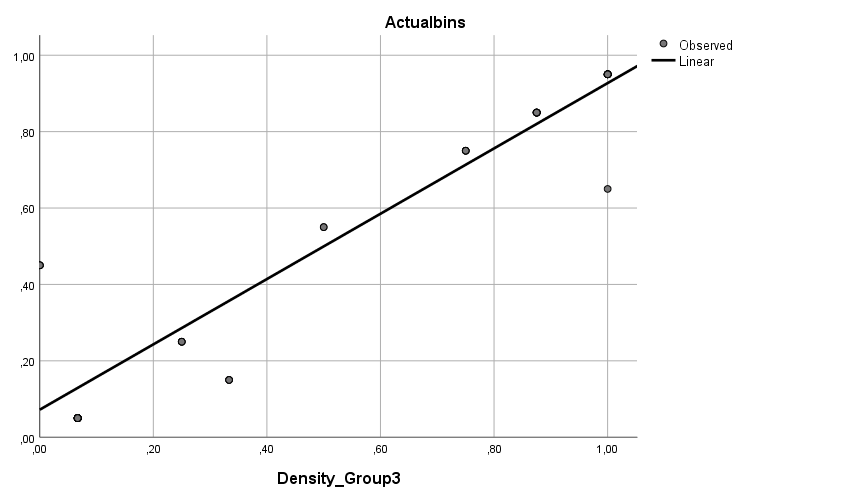


Best fitting line

R^2^=.871

**Actual probability**

**Predicted probability**

**Figure S3c. A calibration plot of the final (combined-domains) model in the validation dataset**
