# Supplementary material for: Screening for Depression in Daily Life: Development and External Validation of a Prediction Model Based on Actigraphy and Experience Sampling Method
Source: J Med Internet Res. 2020 Dec 1;22(12):e22634. doi: 10.2196/22634 (PMC7894744; doi:10.2196/22634)
Supplement: Multimedia Appendix 6 [file jmir_v22i12e22634_app6.docx]

# **Table S2. Sensitivity, specificity, and cut-off score for the ESM model, the actigraphy model and the final (combined-domains) model in the development dataset**

| **Cut-off score** | **True Positives (n)** | **True Negatives (n)** | **False Positives, n (%)** | **False Negatives, n (%)** | **Sensitivity (%)** | **Specificity (%)** | **Youden index** |
| --- | --- | --- | --- | --- | --- | --- | --- |
| ESM model | | | | | | | |
| 0.222 | 43 | 76 | 6 (7.3) | 0 (0) | 100 | 92.7 | 0.927 |
| Actigraphy model | | | | | | | |
| 0.151 | 42 | 40 | 42 (51.2) | 1 (2.3) | 97.7 | 48.8 | 0.465 |
| 0.183 | 40 | 43 | 39 (47.6) | 3 (7.0) | 93.0 | 52.4 | 0.454 |
| Final (combined ESM and actigraphy) model | | | | | | | |
| 0.265 | 42 | 79 | 3 (3.7) | 1 (2.3) | 97.7 | 96.3 | 0.940 |

Note: ESM - Experience Sampling Method. Youden index is the sum of sensitivity and specificity minus one, it estimates the probability of an informed decision and defines an optimal cutoff.
