# Supplementary material for: Screening for Depression in Daily Life: Development and External Validation of a Prediction Model Based on Actigraphy and Experience Sampling Method
Source: J Med Internet Res. 2020 Dec 1;22(12):e22634. doi: 10.2196/22634 (PMC7894744; doi:10.2196/22634)
Supplement: Multimedia Appendix 7 [file jmir_v22i12e22634_app7.docx]

**Table S3. Sensitivity, specificity, and cut-off score for the ESM model, the actigraphy model and the final (combined-domains) model in the validation dataset**

| **Cut-off score** | **True Positives (n)** | **True Negatives (n)** | **False Positives, n (%)** | **False Negatives, n (%)** | **Sensitivity (%)** | **Specificity (%)** | **Youden index** |
| --- | --- | --- | --- | --- | --- | --- | --- |
| ESM model | | | | | | | |
| 0.337 | 23 | 22 | 4 (7.8) | 2 (3.9) | 92.0 | 84.6 | 0.766 |
| Actigraphy model | | | | | | | |
| 0.472 | 18 | 16 | 10 (19.6) | 7 (13.7) | 72.0 | 61.5 | 0.335 |
| Final (combined ESM and actigraphy) model | | | | | | | |
| 0.571 | 22 | 24 | 2 (3.9) | 3 (5.9) | 88.0 | 92.3 | 0.803 |

Note: ESM - Experience Sampling Method. Youden index is the sum of sensitivity and specificity minus one, it estimates the probability of an informed decision and defines an optimal cutoff.
