# Supplementary material for: Screening for Depression in Daily Life: Development and External Validation of a Prediction Model Based on Actigraphy and Experience Sampling Method
Source: J Med Internet Res. 2020 Dec 1;22(12):e22634. doi: 10.2196/22634 (PMC7894744; doi:10.2196/22634)
Supplement: Multimedia Appendix 8 [file jmir_v22i12e22634_app8.docx]

**Table S4. Quantitative comparisons of the articles referred to in the discussion**

| Ref. number | First author | Year | Number of participants | Sample description | Outcomes | Predictors | Findings |
| --- | --- | --- | --- | --- | --- | --- | --- |
| 21 | Hori | 2016 | 40 | 20 MDD individuals,  20 non-depressed individuals | SCID-I | Actigraphy-based (MESOR, amplitude, acrophase), sleep diary | Lower MESOR and more fragmented sleep significantly predicted MDD diagnosis |
| 30 | Maglione | 2014 | 3020 | Community-dwelling older women | GDS | Actigraphy-based (MESOR, amplitude, acrophase, left half-deflection, right half-deflection, pseudo F-statistic) | More depressive symptoms associated with more desynchronization of RAR |
| 31 | Difrancesco | 2019 | 359 | 93 current MDD/anxiety individuals, 176 remitted MDD/anxiety individuals,  90 non-depressed/no anxiety individuals | CIDI | Actigraphy-based (SD, SE, relative amplitude, MSF, gross motor activity, and MVPA), self‐reported insomnia rating scale, SD, MSF, metabolic equivalent total, and MVPA | Reduced activity level and daily rhythm disturbances among individuals with depressive and anxiety disorders |
| 32 | Kim | 2019 | 47 | Community-dwelling elderly | SGDS-K, K-HDRS | ESM (mean score),  Actigraphy-based (activity level, SE, light exposure) | Low level of daytime activity, lower SE, and higher levels of ambient light exposure in depressed individuals |
| 58 | de Jonge | 2017 | 107 | Remitted MDD individuals | SCID-I, IDS-SR | PANAS (PA and NA), VAMS (sad mood) | Less PA and more NA predicted increased depressive symptomatology six months later |
| 59 | Wichers | 2010 | 83 | Women with a history of MDD | SCID-I, SCL-90R | ESM (stress-sensitivity, reward experience, NA, PA), EPI-E | Reward experience and NA variability predicted future negative affective symptoms (at 3, 6, 9, and 12 months follow-up) |
| 60 | Wichers | 2012 | 47 | MDD individuals | HDRS | ESM (NA, PA) | Future response to treatment (up to 6 months) was associated with altered baseline NA–PA dynamics |
| 61 | Geschwind | 2011 | 49 | MDD individuals | HDRS | ESM (NA, PA) | Early change in PA rather than NA predicted response to treatment |
| 62 | Cohen | 2019 | 591 | Adolescents without a history of MDD | K-SADS-PL, CDI | ALEQ (adversity exposure, academic and social impairment), PA, NA | Self-reported rumination, social/academic impairment, and NA best predicted first depression onsets in youth |
| 63 | Andrews | 2017 | 40 | Community-dwelling older adults | GDS | Self-reported mood items ‘happy’, ‘sad’, ‘tired’, ‘alert’, ‘relaxed’, ‘hungry’ | Items ‘sad’ and ‘tired’ are sensitive measures to predict future depression (10 weeks follow-up) |
| 67 | Todder | 2009 | 54 | 27 MDD individuals, 27 non-depressed individuals | SCID-I, HDRS | Actigraphy-based (level and quantity of physical activity) | Day-time and night-time motor activity captures distinct patterns of motor activity as well as clinical course of depression |
| 68 | Smagula | 2015 | 2933 | Community-dwelling older men | GDS | Actigraphy-based (acrophase, up-MESOR, down-MESOR, and pseudo-F statistic | Low activity rhythm height/robustness with normal timing indicates depression risk; late or combined early/compressed/dampened activity rhythms contributes to depression symptom development |
| 71 | Lyall | 2018 | 91105 | General population | MHQ | Actigraphy-based (relative amplitude) | Reduction in relative amplitude was associated with increased risk of lifetime MDD and lifetime BD, greater mood instability, higher neuroticism scores, more subjective loneliness, lower happiness, lower health satisfaction, and slower reaction times |
| 72 | Nebes | 2009 | 157 | Community-dwelling elderly | GDS | PSQI (self-reported sleep latency, duration, and efficiency) | Poor sleep was associated with increased depressive symptomatology but only for functional symptoms and not for mood |

**Abbreviations**. **ALEQ** - the Adolescent Life Event Questionnaire; **BD** – Bipolar Disorder; **CDI** - the Children’s Depression Inventory; **CIDI** - the Composite International Diagnostic Interview; **EPI-E** - the Neuroticism–Extraversion subscale of the Eysenck Personality Scale; **ESM** – Experience Sampling Method; **GDS** - Geriatric Depression Scale; **HDRS** - the Hamilton Depression Rating Scale; **IDS-SR** - the Inventory of Depressive Symptomatology – Self Report; **K-SADS-PL** - Mood disorders section of the Schedule for Affective Disorders and Schizophrenia for School Age Children; **MDD** – Major Depressive Disorder; **MESOR** - Midline Estimating Statistic Of Rhythm; **MHQ** - an Online Mental Health Questionnaire (questions derived from the CIDI); **MSF** - mid sleep on free days; **MVPA** – moderate-to-vigorous physical activity; **NA** – negative affect; **PA** – positive affect; **PANAS** - the Positive and Negative Affect Schedule; **PSQI** - Pittsburgh Sleep Quality Index; **RAR** – rest-activity rhythm; **SCID** - Structured Clinical Interview for DSM-IV; **SCL-90R** - the Symptom Checklist; **SD** - sleep duration; **SE** - sleep efficiency; **SGDS** - Short Geriatric Depression Scale; **VAMS** - Visual Analogue Mood Scale
